# 

**Published:** 1913-07-26

**Authors:** 


### Buildings Contemplated.

Fazakerley.—The Liverpool Corporation has decided to erect a sanatorium on a portion of the hospital estate at Fazakerley.

Garstang.—The Rural District Council has decided to obtain tenders for an isolation hospital.

Halifax.—An inquiry has been held by the Local Government Board into an application of the Town Council for sanction to a loan for the purchase of the Green Lane Hall Estate, Shelf, and for the building thereon of a sanatorium. The scheme is to be carried out under the supervision of the Borough Engineer, Mr. J. Lord.

Hampton.—The new St. Mary's Cottage Hospital in Sundury Road, Hampton, has been formally opened.

Hindley.—The Urban District Council has resolved to provide additional hospital accommodation at a cost of £4,000.

Ladysbridge.—The plans submitted by the Banffshire District Lunacy Board for the enlargement of the mental hospital have been approved by the General Lunacy Board.

Llanelly.—The Guardians have issued instructions for the preparation of plans for an isolation hospital. It is estimated that the cost will be £10,442.

New Brunswick.—It is estimated that the contemplated improvements to the General Public Hospital at St. John will cost £20,000.

Newtown.—The Montgomeryshire County Council has decided to abandon a scheme for adapting the Forden Workhouse as a mental hospital, and to erect a new building at a cost of £57,000.

St. Austell.—A scheme has been adopted by the Guardians for the reconstruction of the infirmary at an estimated cost of £3,000.

Stannington.—A scheme to raise £2,000 for erecting a farmhouse in connection with the sanatorium is under consideration.

Yarmouth.—Messrs. Alley and Haward have been instructed by the Guardians to prepare an estimate of the cost of providing ten bedrooms, a sitting room, and kitchen to be added to the existing nurses' home.

### LITERARY INTELLIGENCE.

An important new work is about to be published, entitled "Researches on Rheumatism," by Dr. F. J. Poynton and Dr. Alexander Paine, being a collection of the chief papers bearing upon a research on the subject of rheumatism which has extended over a period of fifteen years. At the conclusion of the volume the bearing of these investigations upon clinical medicine and public health is considered in a special article. The work, which is to contain 106 black-and-white plates and a frontispiece in colour, will be published by Messrs. J. and A. Churchill, of 7 Great Marlborough Street, W. The same firm has nearly ready the eleventh edition of Swayne's "Obstetric Aphorisms," which is now edited by Dr. W. C. Swayne, Professor of Obstetrics in the University of Bristol; also the seventh edition of "The Microtomist's Vade-Mecum," by Mr. A. B. Lee; the sixth edition of the late Professor Campbell Brown's "Practical Chemistry," edited by Dr. Bengough; and the third edition of "A Text-Book of Physics," edited by A. Wilmer Duff.

### Personalia.

Mr. Robert Wyvill Barclay has been elected a governor of Guy's Hospital.

Sir George Riddell has kindly consented to occupy the chair on the occasion of the seventy-fourth anniversary festival of the Newsvendors' Benevolent and Provident Institution, which will be held at De Keyser's Royal Hotel, Whitefriars, in the City of London, on Monday, November 3 next.

Sir Frederic Eve has been elected a member of the executive committee of the Imperial Cancer Research Fund, and Mr. R. Clement Lucas has been appointed the representative of the Royal College of Surgeons on the Council of the Queen Victoria's Jubilee Institute for Nurses. Mr. Hugh Wilde was appointed solicitor to the College in succession to his father, Mr. Ernest J. Wilde, resigned.

A gap among workers on behalf of charitable institutions has been left by the recent decease, at the age of only forty-nine, of Mrs. Gertrude Thomas, of Ruabon, North Wales. Mrs. Thomas was a loyal supporter of the Carmarthen Infirmary, and year after year helped to organise the annual ball for its benefit. Her grandfather on her mother's side, Dr. James Rowlands, was consulting surgeon of that institution for a considerable number of years. The drug business founded by her grandfather at Carmarthen was carried on by her father, Mr. William de Gruchy Warren. Another branch of the business was carried on by three generations of the Warren family in succession at Bristol, and is now in the hands of Messrs. Evans, Gadd, and Co., Ltd., who purchased it at the close of 1897, on the retirement of Mr. Algernon Warren, brother of the President of Magdalen College, Oxford.

### Appointments.

Mr. G. H. Stevenson has been appointed fifth assistant medical officer at Bexley Mental Hospital.

Mr. D. Isaacs, M.R.C.S., L.R.C.P., has been appointed assistant medical officer at the Shoreditch Infirmary.

Mr. Kenelm H. Digby, M.B., B.S., F.R.C.S., has been appointed Professor of Anatomy at the University of Hong-Kong.

Mr. Hedley Boyers, of Dr. Steevens' Hospital, Dublin, has been appointed assistant house surgeon at the West Bromwich and District Hospital.

Mr. P. S. P. Langton has resigned his appointment as fifth assistant medical officer at Claybury Mental Hospital, and the sixth assistant has been promoted a grade.

Miss H. Patricia Barnes, M.B., B.Sc.(Lond.), has been appointed house physician, and Mr. John Douglas Cherry, L.R.C.P.I., L.R.C.S.I., second house surgeon at the East Suffolk and Ipswich Hospital, Ipswich.

Mr. A. Richardson, M.B., B.S.(Lond.), F.R.C.S., of Park Square, Leeds, who recently retired from the position of resident surgical officer at the Leeds General Infirmary, has been appointed by the University of Leeds to be surgical tutor in connection with the Medical School, and the weekly board of the General Infirmary have appointed him surgical registrar.

Guy's Hospital.—Mr. J. Lindsay Locke, M.B., Ch.B.(Edin.), has been appointed senior medical radiographer; Mr. H. T. Depree, dental anæsthetist; Mr. A. J. Clark, M.B.(Cantab), M.R.C.P.(Lond.), lecturer in pharmacology; Dr. Fawcett, lecturer in medicine; Mr. Chapple, demonstrator in charge of revision classes in midwifery; Mr. Davies-Colley, curator of Gordon Museum; Dr. Cameron, pathologist; and Dr. Hunt, demonstrator of medical pathology.

### Gifts and Bequests.

The Samaritan Free Hospital for Women, Marylebone Road, London, N.W., has received £25 from the Co-operative Wholesale Society, Ltd., Manchester.

King Edward's Hospital Fund for London has received £2,000 from the Drapers' Company, being the half-yearly payment of their annual subscription of £4,000.

The sum of £100, obtained for one rose on Alexandra Day by Lady Byron, is to be sent to the Hospital for Ladies of Limited Means, Osnaburgh Street, which was started some years ago by the late Rev. Arthur Brinckman.

Mr. Alfred King, of Worthing, has directed that the residue of his property should accumulate during the life of his wife, and on her death and the falling in of the several annuities he left the reversion of his property to Guy's Hospital.

The late Mrs. Anne Jane Twyford has bequeathed £500 to the Liverpool Convalescent Institution at Woolton, free of duty. The Liverpool Blue Coat Hospital, the Children's Rest at Wavertree, the School for the Blind (Children's Branch), Wavertree, and two non-medical charities will each receive one-fifth of the residue, which, as the net estate has been sworn at £81,000, should be a large sum.

The following are among the additional amounts recently received at the Mansion House in respect of collections on Hospital Sunday: West London Synagogue, £289; Holy Trinity, Sloane Street, £285; Christ Church, Mayfair, £133; St. Paul, Avenue Road, N.W.. £117; and Victoria Park Christian Evidence Association, £100. The total sum available for distribution up to Saturday, July 12, is about £52,000.

The committee appointed to organise the League of Mercy concert on May 24 last has granted out of the small surplus £25 to the Actors' Benevolent Fund and £20 to the Newspaper Press Fund. The remaining balance was allocated to the Samaritan Wing of the League, which endeavours to help its poorer members by providing them with assistance in obtaining admission to convalescent homes, and surgical-aid appliances and spectacles.

Mr. Alfred Hugh Harman, of Haslemere, has left his freeholds on the Southville Park Estate, Dulwich, S.E., for the use of King Edward's Hospital Fund for London; freeholds at Harlesden and his shares in the British Law Fire Insurance Company, Ltd., to the London Orphan Asylum, Watford; to the Three Counties Nursing Association, Shottermill, Surrey, £1,000 Debentures of Drummond Brothers, Ltd.; to the Haslemere Cottage Hospital £1,000 Debentures and all his Preference Shares in Drummond Brothers, Ltd.; £5,000 each to Dr. Barnardo's Homes and the City of London Hospital for Diseases of the Chest; £2,000 each to the Discharged Prisoners' Aid Society; the Institution for Poor Gentlewomen, 20 Great Portland Street, W., and the British Home for Incurables, Streatham; £1,000 each to the Charity Organisation Society, the After-Care Association, the Royal Association in Aid of the Deaf and Dumb, and St. John's Hospital, Leicester Square.

### Obituary.

### HOSPITAL CHAPLAIN FOR TWENTY YEARS.

While on a visit to Taunton on Friday last the death occurred of the Rev. Edward A. Ducket, vicar of East Pennard, at the age of fifty-three years. Mr. Ducket, who was the son of a Yarmouth doctor, was curate of Tiverton for seven years, and vicar of Taunton from 1890 to 1911, during which twenty years he was chaplain of the Taunton and Somerset Hospital.

### DOCTORS' WILLS.

Mr. Arthur Edward Barlow, M.B., C.M., M.R.C.S., of Leicester, left estate valued at £52,580 gross, with net personalty £18,580.

Dr. Lyttelton Stewart Forbes Winslow, LL.D., D.C.L., M.B., M.R.C.P., of Devonshire Street, Portland Place, W., who died on June 8, aged sixty-nine, has left estate valued at £27 11s. 5d.

### An Appeal.

A nurse from one of the loneliest and most inaccessible islands of the Aran group in Galway Bay writes to ask for a gramophone to cheer the lonely islanders in the winter months. A second-hand gramophone would be acceptable so long as it is in good condition. The winter in these islands is exceptionally wild, and the loneliness almost unbearable, so that any of our readers who will respond to this appeal will have the satisfaction of knowing that they are bringing joy and pleasure to a very worthy little band of folk whose lives are entirely without any amusement. Replies should be sent to The Editor, The Hospital, at 28 Southampton Street, Strand, London, W.C., and marked "Gramophone" in left-hand top corner of envelope.

